# Multi-Order Investigation of the Nonlinear Susceptibility Tensors of Individual Nanoparticles

**DOI:** 10.1038/srep25415

**Published:** 2016-05-03

**Authors:** Cédric Schmidt, Jérémy Riporto, Aline Uldry, Andrii Rogov, Yannick Mugnier, Ronan Le Dantec, Jean-Pierre Wolf, Luigi Bonacina

**Affiliations:** 1 Université de Genève, GAP-Biophotonics, 22 chemin de Pinchat, Carouge, 1211 Geneva 4, Switzerland; 2 Univ. Savoie Mont Blanc, SYMME, F-74000 Annecy, France

## Abstract

We use Hyper Rayleigh Scattering and polarization resolved multiphoton microscopy to investigate simultaneously the second and third-order nonlinear response of Potassium Niobate and Bismuth Ferrite harmonic nanoparticles. We first derive the second-to-third harmonic intensity ratio for colloidal ensembles and estimate the average third-order efficiency of these two materials. Successively, we explore the orientation dependent tensorial response of individual nanoparticles fixed on a substrate. The multi-order polarization resolved emission curves are globally fitted with an analytical model to retrieve individual elements of susceptibility tensors.

The great richness and potential for new applications of nonlinear optics resides in the multiple different signals that can be generated simultaneously. For example, second order susceptibility response, *χ*^(2)^, already accounts for second harmonic generation, frequency mixing, and optical rectification. Increasing nonlinear order corresponds to a further increase in the number of different signals that can be accessed and prospectively exploited for sensing or imaging^1^. However, the simultaneous collection of emissions stemming from different *χ*^(*n*)^ orders from a single object has been scarcely reported to date, due to several complications associated with this measurement. On one hand, for macro- and microscopic objects, phase-matching and sample transparency restrictions come into play and prevent efficient signal build up and propagation in the far field. On the other hand, for nanoscopic systems, where these constraints are absent or relaxed, the simultaneous detection of different *χ*^(*n*)^ responses is commonly prevented by their extremely large intensity differences.

An interesting model system for exploring these phenomena is represented by Harmonic Nanoparticles (HNPs), which have been introduced as bio-labelling agents for multiphoton microscopy approximately ten years ago^2^. Differently from the case of plasmonic nanoparticles^3^,^4^,^5^, harmonic generation by HNPs to be efficient should not be excited at specific resonances^6^. Moreover, the presence of even harmonics is not associated with symmetry breaking at particles surface like in the case of metal particles but directly to the noncentrosymmetric character of their lattice structure, at least for sizes >20 nm^7^. Generally, HNPs possess very high average second-order nonlinear coefficients 〈*χ*^(2)^〉 up to 160 pm/V^8^,^9^. Moreover, for this category of materials, the average third order response is also expected to be large and scaling as |*χ*^(3)^| ∝ |*χ*^(2)^|^2^ 10.

Very recently, a few authors have observed the simultaneous emission of second and third harmonic (SH, TH) by individual HNPs in microscopy^11^,^12^. The simultaneous detection of these two signals can be exploited for sensing^13^ and imaging^14^ to increase selectivity against background endogenous signals in particular collagen (SH) and lipids (TH)^15^. Clearly similar signal intensity at the two nonlinear orders is mandatory to detect SH and TH emission with the same set-up under identical excitation intensity. On the other hand, the tunability of the response of HNPs is of particular interest for performing measurements in the new short-wave excitation windows recently highlighted where efficient labels are still lacking^16^. An aspect which is still pending after these first works is a sound estimation of TH generation efficiency with respect to SH, which is in contrast established for most materials^8^,^9^,^17^. Note that experimentally the TH to SH signal ratio is modulated by the excitation intensity, *I*, because of the different nonlinear dependence of the two signals: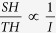. Moreover, although harmonic emission by HNPs has been reported as largely independent from excitation wavelength, one could expect a modulation of the emission efficiency associated with the electronic properties of the material for TH generated at the edge of the transparency range^18^. Another element of interest is related to the tensorial properties of the nonlinear susceptibilities associated with both harmonic emissions. The latter could be in principle investigated using the tools developed for SH by the Zyss group^19^ and successively applied by several authors for interpreting the polarization-resolved SH emission of subfocal structures in microscopy^20^,^21^,^22^.

In the following, we focus on two noncentrosymmetric metal oxide HNPs which have already been investigated in their nanometric form: Potassium Niobate (KNbO_3_, KN in the following) and Bismuth Ferrite (BiFeO_3_, BFO). The former, has the advantage to be a widespread nonlinear medium for laser applications well characterized in its bulk form in terms of *χ*^(2)^ 23 and with some *χ*^(3)^ tensor elements available in the literature^24^. BFO is, together with BaTiO_3_^18^, considered the most promising material for bio-imaging applications, thanks to its high nonlinearity and magnetic properties^9^, but also because of its noteworthy biocompatibility^25^.

## Experimental Methods

KN particles produced by sol-gel method were kindly supplied by FEE GmbH (Germany). BFO were synthesized by an auto-combustion process by FEE GmbH and provided in stable colloidal suspensions by TiBio SA (Switzerland). Typical concentrations of 0.1 mg/ml were obtained for both samples after sonication and sedimentation^9^. According to Dynamic Light Scattering (DLS), HNPs size was estimated at about 100 nm.

A Hyper Rayleigh Scattering (HRS) set-up^26^ was used to measure the SH and TH scattering of the particle suspensions. A vertically polarized YAG laser (*Wedge HB*, Bright solutions, pulse width 1 ns) is focused by a 20 cm focal length lens into a UV fused quartz cuvette containing the sample. SH and TH scattering is collected perpendicularly to the fundamental beam thought a short pass colored glass filter and an interferometric filter (at 532 nm and 355 nm for SH and TH, respectively) placed in front of a photomultiplier. A half-wave plate associated with a polarizer cube is used to adjust the incident power. Maximum mean power was set to 220 mW during the experiments leading to 11 GW/cm^2^ intensity at the focus of a *f* = 20 cm lens. Note that in our set-up no polarization analyser is present in front of the detector as the application of HRS was limited to the derivation of orientation-averaged quantities.

**Figure 1:**
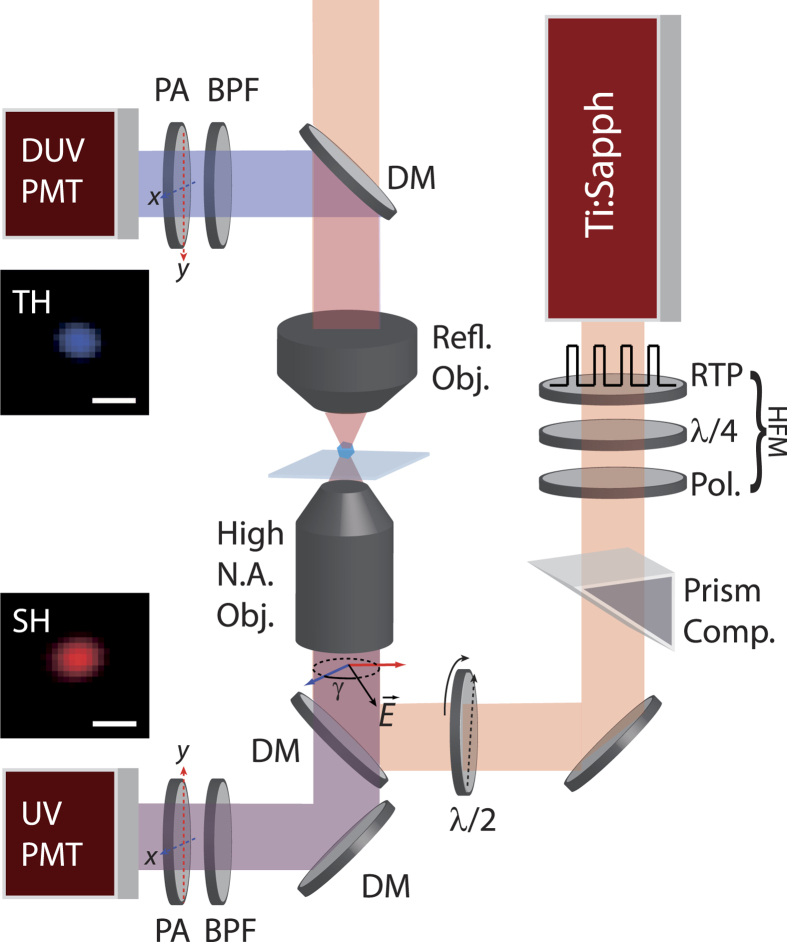
Schematics of the multi-harmonic polarization-resolved set-up. HFM: high frequency modulation module. DM: dichroic mirror. BPF: bandpass filter. In the two insets we present actual images of a single HNP fixed on the substrate acquired simultaneously at the SH and TH. The scalebar corresponds to 1 *μ*m.

For the polarization response of individual HNPs, the 80 MHz output of a Ti:Sapphire oscillator centred at *λ*_0_ = 790 nm (*Femtosurce*, Femtolasers) is modulated in amplitude at 4.88 MHz by a high frequency module (HFM in Fig. 1) comprising of a RTP electrooptic crystal (*Qubig*, Germany) driven by the RF output signal generated by a lockin amplifier (*SR844*, Stanford Research Instruments), a quarter wave plate, and a polarization analyser. The pulses are then anti-chirped by a prism compressor (*BOA*, Swamp Optics) to pre-compensate for dispersion through the optical elements. The linear laser polarization is freely modified by a *λ*/2 plate mounted on a motorized rotation stage. The beam is finally injected into a high N.A. objective and focused onto a single HNP, with a peak intensity of the order of 10 GW/cm^2^. In this case the sample was prepared by casting a drop of HNPs solution and letting it dry prior to measurements, as previously reported^9^,^20^. The position of the nanocrystal in the focus is maximized by a three dimensional piezo-stage (Physik Instrumente, Germany). The SH emission by the HNP is epi-collected using the same objective, spectrally filtered by a combination of two dichroic mirrors (DM) and a bandpass filter centred at *λ*_0_/2 and detected by a photomultiplier. The signal is finally processed by the high frequency lockin at the HFM driving frequency. The TH emission is forward collected by Schwarzschild objective (*ReflX*, Edmund Optics), spectrally filtered and detected by a dedicated photomultiplier. Note that the use of a reflective objective allows to circumvent the issue of transmission cut-off of collection optics at the TH of Ti:Sapphire laser, a problem which can alternatively be solved using a 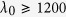 nm ultrafast laser^11^,^12^,^13^,^14^,^27^. The polarization of both SH and TH signals is finally analysed by a Glan-Thompson polarizing cube placed right in front of the detector. The integration time on the lock-in was set to 100 ms per data point.

Multiple fitting procedures were run on a standard personal computer using the Global Analysis Package of *IgorPro v.637* by Wavemetrics.

## Results and Discussion

### HRS on colloidal ensembles

**Figure 2:**
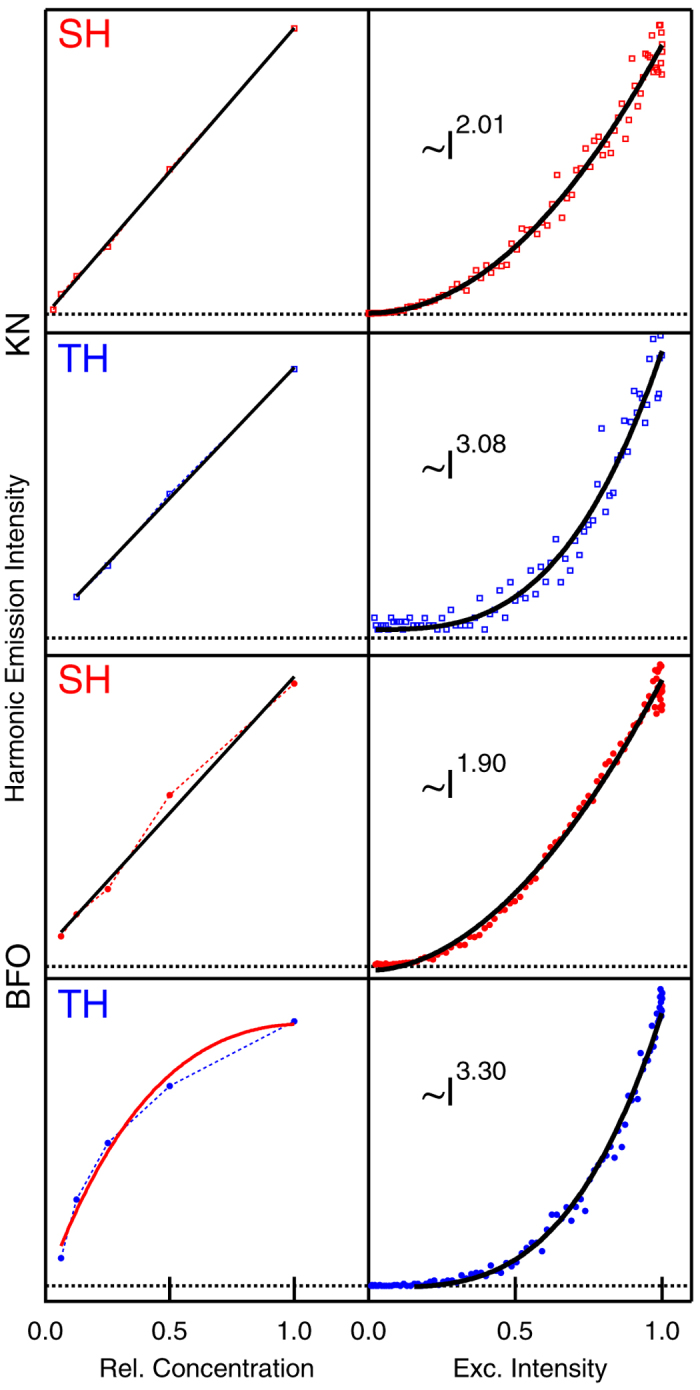
HRS results. Left column. Concentration dependence of SH and TH signals from colloidal solutions of KN and BFO HNPs. Solid lines: linear fits except for BFO TH, fitted by *A* ⋅ *Ne*^−*αN*^, where *A* is a proportionality constant, *N* is the relative concentration and *α* a factor accounting for linear extinction by HNPs at the TH frequency. Right column: Intensity dependence for SH and TH emission. Solid lines: results of the fits of the experimental curves by the expression *A* ⋅ *I*^*n*^, the fitted values for *n* are reported.

In the right column of Fig. 2, we report the SH and TH signal strength as a function of excitation intensity *I* from HRS along with their fits by a power law. As expected, the curves are proportional to *I*^*n*^ with 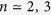 for SH and TH, respectively. The corresponding emission spectra were also acquired setting a monochromator in front of the photomultiplier, indicating pure SH and TH signatures with no background (Figure S1). The SH and TH scattered intensities were also measured as a function of the particle relative concentration (Fig. 2, left column). In this case, SH and TH signals are linearly proportional to the number of probed particles for low particles concentration. At higher concentrations, we observe a clear deviation from this trend for BFO TH. Such a finding can be ascribed to the effect of sample linear extinction, as modelled in the fit curve superimposed to the data^26^.

Considering exclusively the linear part of the intensity versus concentration curves and taking into account the different collection efficiencies for the SH and TH wavelengths, the SH/TH ratio could be estimated at 30 for BFO and 322 for KN for 11 GW/cm^2^ excitation intensity. Based on this ratio, we can calculate the orientationally averaged third order efficiency 〈*χ*^(3)^〉 (at 1064 nm) for both nanomaterials (Equation S4). The value extracted for KN is 〈*χ*^(3)^〉 = 1 × 10^−19^ m^2^/V^2^. For a reference, Bosshard *et al.* reported 
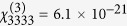
 m^2^/V^2^ from bulk measurements at 1318 nm and they extrapolated a value of 1.2 × 10^−20^ m^2^/V at 1064 nm using a model which does not account for absorption effects^24^. For BFO, the estimated 〈*χ*^(3)^〉 is 5.5 × 10^−18^ m^2^/V ^2^. We remark that for typical oxide materials, the measured values are fairly high but are probably related to electronic resonance at TH frequency which falls within the absorption band in particular for BFO. Moreover, TH signal can originate both from a *pure* third order process as well as from cascaded second order processes. The two mechanisms cannot be easily distinguished but the relative contribution of each should scale as (*χ*^(3)^/(*χ*^(2)^)^2^)^2^ 28. It can be estimated at least for KN, whose *χ*^(2,3)^ values are known^24^, that cascaded process is on average less important. In terms of absolute efficiency comparison, useful for translation to TH microscopy applications, Boyd *et al.* in a very recent paper^29^ critically analysed the third order nonlinear optical susceptibility values reported for gold in the literature, which spans from 10^−14^ to 10^−19^ m^2^/V^2^.

### Polarization-resolved microscopy on fixed HNPs

#### KN HNPs

**Figure 3:**
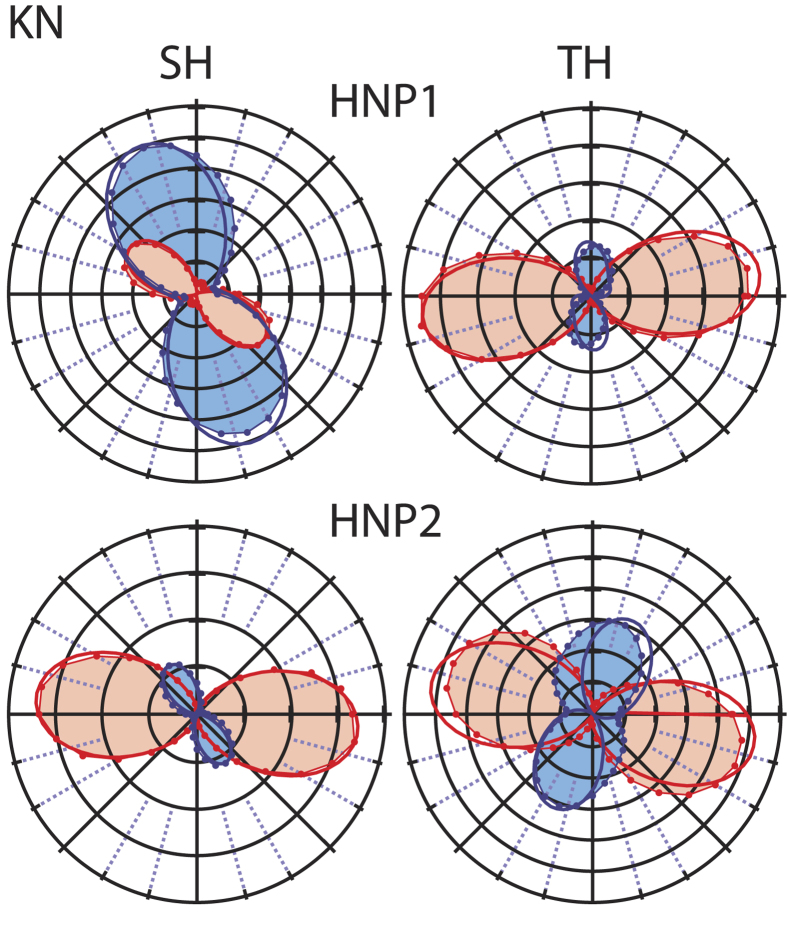
Polarization-resolved emission plots at the SH and TH for two different sub-diffraction limited KN HNPs. Dots/shaded areas: experimental curves obtained with horizontal (red) and vertical (blue) analyzer. Thick lines: results of model fitting. Euler angles for HNP1 (upper row): *ϕ* = 111°, *θ* = 173°, *ψ* = 136°. HNP2 (lower row): *ϕ* = 159°, *θ* = 117° and *ψ* = 17°.

In the following, we focus on the tensorial character of the nonlinear signals generated by HNPs assuming only pure TH contribution. Figure 3 reports the polarization plots at both SH and TH obtained for two KN individual nanoparticles. The dots represent the experimental data points obtained for a given angle *γ* of the linearly polarized excitation laser and for horizontal (red) and vertical (blue) position of the polarization analysers (see Fig. 1). In general, one can appreciate the mirror symmetry of the polar plots obtained and the presence of nodal points of very low (practically zero) intensity along defined axes, pointing to the monocrystalline nature of the particles under investigation^20^,^30^. This finding is also in line with the sub-diffraction limited dimensions of the nanoparticles chosen for this analysis, as reported in the insets of Fig. 1 showing an individual HNP imaged simultaneously at the SH and TH. For each HNP the SH and TH curves possess a marked distinct appearance, reflecting substantial difference in the tensorial response among the two nonlinear orders. To investigate this essential aspect, which eventually modulates the SH/TH ratio of individual HNPs, we proceeded in modelling both responses. In the following, the excitation beam is supposed at normal incidence on the sample, with only two in-plane components, *E*_*x*_ = *E*cos(*γ*) and *E*_*y*_ = *E*sin(*γ*), ignoring the longitudinal field component similarly to previous works by ours and other groups^17^,^20^,^21^,^22^,^30^. The second order polarization 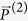 is then written as


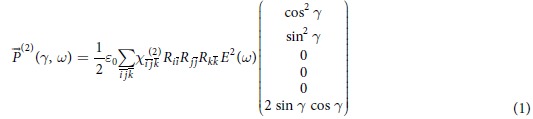


**Figure 4:**
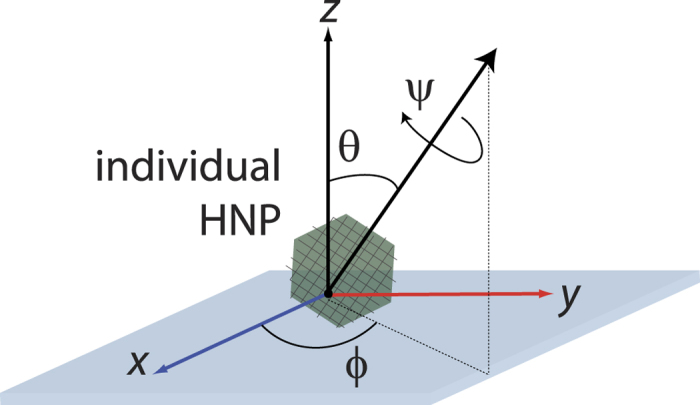
Definition of Euler angles. {*ϕ*, *θ*, *ψ*} define the orientation of the crystal axis of a single HNP fixed on a microscope substrate with respect to the laboratory frame {*x*, *y*, *z*}.

In the equation, three successive rotation operations 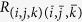 are applied to express in the laboratory frame the second order susceptibility tensor 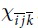 given in the crystal frame. These transformations are functions of the Euler angles {*ϕ*, *θ*, *ψ*} defining the orientation of the crystal axis with respect to the laboratory frame {*x*, *y*, *z*} as in Fig 4.

For modelling the SH response, the *χ*^(2)^ tensor was simplified to a 6 × 3 matrix using Voigt notation^1^. For the *mm*2 point group symmetry associated with KN, 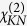 features 5 nonzero independent elements^23^:


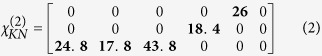


During the fitting procedure, all 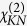 elements were kept fixed and the Euler angles let free to vary, to retrieve the orientation of each HNP, consistently with the procedure applied in previous works^8^,^17^,^20^,^30^. As one can see from the final plots in Fig. 3, the agreement with the fits is rather good, even if in the simplified model applied here the corrections for large N.A. collection are not accounted for to reduce the computational complexity of the fitting expression. The orientation angles retrieved for these two exemplary HNPs are reported in the caption.

The approach was successively extended to account also for third order polarization. Briefly, keeping the same formalism introduced above, the polarization vector is now written as


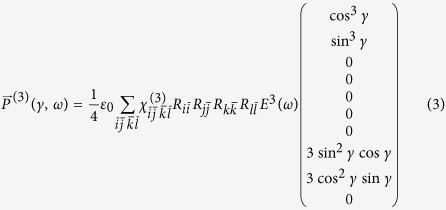


To our best knowledge, there exists no complete quantitative description of all the 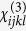 tensor elements of KN, while the overall symmetry of the tensor (and the useful mapping procedure from the tensor to the Voigt matrix) can be found in the work by Yang and Xie^31^. We therefore used the three diagonal elements 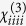 measured by Bosshard *et al.* on bulk samples^24^ and let the fitting algorithm retrieve the three missing ones according to the *mm*_2_ symmetry of the material. Note that to over-determine the system to be solved and increase the statistical significance of the analysis, the TH responses of two particles were simultaneously fitted keeping the Euler angles retrieved at the SH harmonic as local fixed parameters, and the unknown 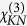 tensor elements as global parameters to be determined. Although slightly less congruent with the experimental curves than in the case of SH, the fitted curves reproduce fairly well the general trends of the third order nonlinear response. The final 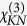 assumes the following form, where the elements kept fixed in the fitting are printed in bold font:


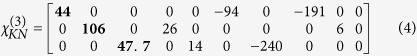


An import *caveat* applies here: the bold values - although absolute - were determined by Bosshard at a different wavelength than the one used in this work. Therefore, although the relative relationships among the tensor elements are informative for 800 nm excitation, their absolute values including the elements in bold font might be different.

#### BFO HNPs

**Figure 5:**
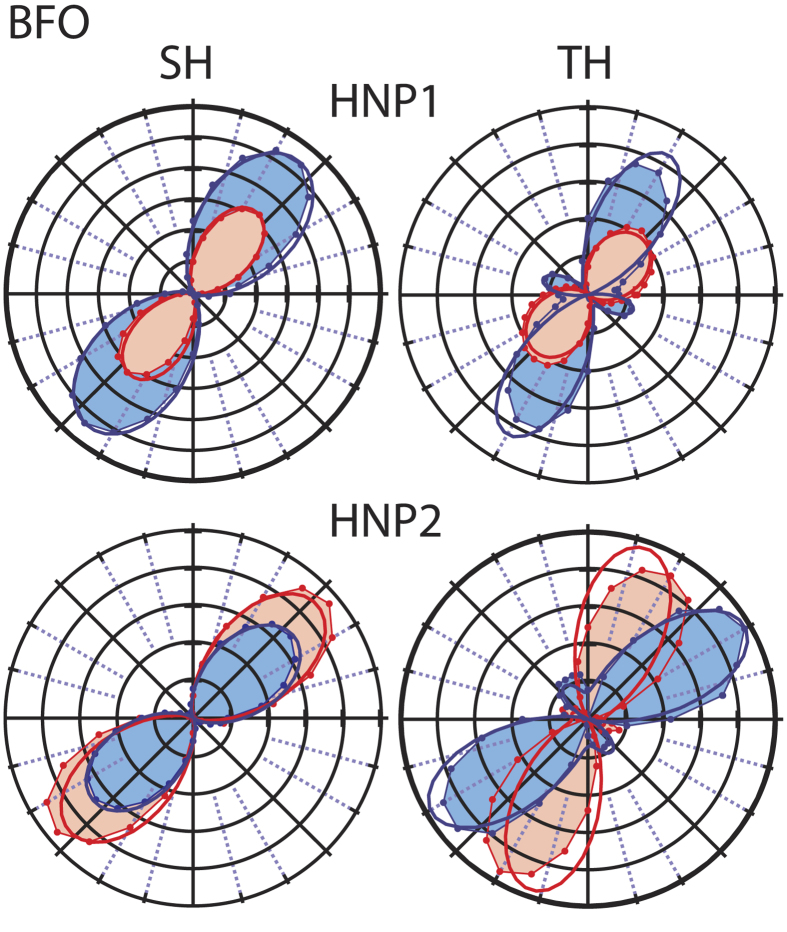
Polarization-resolved emission plots at the SH and TH for two different sub-diffraction limited BFO HNPs. Dots/shaded areas: experimental curves obtained with horizontal (red) and vertical (blue) position of the analyzer. Thick lines: results of model fitting. Euler angles for HNP1 (upper row): *ϕ* = 42°, *θ* = 108°, *ψ* = 54°. HNP2 (lower row): *ϕ* = 52°, *θ* = 81° and *ψ* = 49°.

We then repeated the procedure just outlined for analysing the multi-order polarization plots of BFO HNPs. The data are reported in Fig. 5. Again the responses associated with the overall features of the curves indicate the monocrystalline characteristics of the selected HNPs, and the two harmonic orders yield different shapes. The point group of BFO is 3*m* which corresponds to 8 nonzero elements (4 independent) for 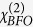 and 13 (6) for 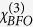. Differently from the case of KN, the information for BFO are already quite scarce and often contradictory for the second order response. As we discussed in a previous publication^9^, this can be associated with the slightly different stoichiometry ensuing from various synthetic protocols proposed in the literature, which can affect the lattice properties. Here we use a set of *relative* 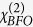 values originally obtained on thin film samples^32^ which we already successfully applied to model the response of different individual BFO HNPs probed by SH microscopy^9^. From this set of relative values and the absolute average second-order 〈*χ*^(2)^〉 determined at 160 pm/V by Schwung *et al.*^9^ by HRS measurements, the non-zero elements of the *χ*^(2)^ BFO tensor were then estimated. Note that without Kleinmann symmetry in the case of 3*m* crystals and for a vertically polarized laser with no analyser in the detection path, 〈*χ*^(2)^〉^2^ can be expressed as by developing HRS full polarization formalism originally introduced for nonlinear molecules^33^,^34^,^35^ to the case of nanocrystals^26^:


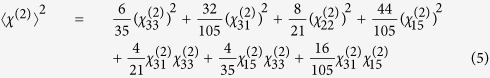


from which we obtained :


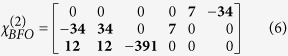


Using this matrix we could determined the angles of various HNPs like the two reported in Fig. 5 and again the quality of the fits is rather good.

For the BFO TH response the situation is more complicated, as only the 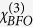 symmetry is known and the position of its nonzero values, but no tensor element was previously identified. We therefore kept as fixed parameters in the global fitting procedure only the Euler angles retrieved at the second order. The results of this fit are again in good agreement with experimental data, and capture correctly the overall features of the traces including the major to minor lobes relative intensity for both particles under study. The 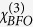 values we retrieved, expressed relatively to the 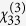 element arbitrarily normalized to 100, are:


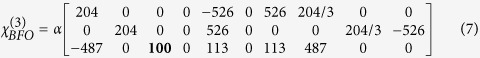


Again, the derivation of an expression equivalent to Eq. 5 for the third order response would allow to determine the absolute tensor values.

## Conclusions

In this work we have performed an investigation of the simultaneous harmonic emission by two different nanomaterials, KN and BFO. To our best knowledge, this represents the first work were the tensorial properties of two different nonlinear orders are simultaneously accessed and modelled. The ensemble measurements by HRS indicate an intensity ratio among the two nonlinear orders of respectively 30 and 322 under 11 GW/cm^2^ intensity at 1064 nm which allowed us to estimate the orientationally averaged third order nonlinear efficiencies for these two nanomaterials (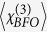 = 5.5 × 10^−18^ m^2^/V^2^ *vs* 
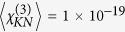
 m^2^/V^2^). For KN the retrieved value is in line with previous works^24^. The smaller value for the SH/TH ratio observed for BFO, together with its much higher absolute intensity for SH (160 pm/V *vs* 16.6 pm/V) is indeed very favourable for setting-up multi-harmonic detection protocols^13^,^14^ which have been demonstrated with the aim of increasing HNP detection selectivity against endogenous signal hindrance. In addition, we have collected and analysed the simultaneous polarization resolved emission at different nonlinear orders highlighting their tensorial character. The traces obtained for KN HNPs are well fitted using a model based on *χ*^(2,3)^ matrices provided in full (second order) or only partially (third order) in the literature. This agreement sets a convenient ground for applying the same approach to BFO which enabled us to derive the relative values of its *χ*^(3)^ matrix. A part from the fundamental interest of the method just demonstrated for investigating the relationship between single elements of susceptibility tensors at different nonlinear orders, this approach can bring benefits to protocols of orientation retrieval of HNPs (*e.g.* particle tracking in biological media^36^ and superresolution^37^) in terms of speed and precision.

## Additional Information

**How to cite this article**: Schmidt, C. *et al.* Multi-Order Investigation of the Nonlinear Susceptibility Tensors of Individual Nanoparticles. *Sci. Rep.* **6**, 25415; doi: 10.1038/srep25415 (2016).

## Supplementary Material

Supplementary Information
