# Supplementary material for: Multi-Order Investigation of the Nonlinear Susceptibility Tensors of Individual Nanoparticles
Source: Sci Rep. 2016 May 3;6:25415. doi: 10.1038/srep25415 (PMC4853726; doi:10.1038/srep25415)
Supplement: Supplementary Information [file srep25415-s1.pdf]

Supplementary material

Multi-Order Investigation of the Nonlinear Susceptibility Tensors of Individual Nanoparticles

CÉDRIC SCHMIDT[1], JÉRÉMY RIPORTO[2], ALINE ULDRY[1], ANDRII ROGOV[1], YANNICK MUGNIER[2], RONAN LE DANTEC[2], JEAN-PIERRE WOLF[1], AND LUIGI BONACINA[1,*]

[1]*Université de Genève, GAP-Biophotonics, 22 chemin de Pinchat, Carouge, 1211 Geneva 4, Switzerland*
[2]*Univ. Savoie Mont Blanc, SYMME, F-74000 Annecy,*
**Corresponding author: luigi.bonacina@unige.ch*

1- Spectra of the scattered light

Spectra of scattered light from BFO and $KNbO_3$ suspensions upon excitation at 1064 nm were obtained by inserting a monochromator in front of the photomultiplier tube. For BFO, pure SH (532nm) and TH (355nm) signals are clearly observed whereas the TH response of $KNbO_3$ was found below the detection threshold when the monochromator is added. The absence of any unexpected background can yet be noticed.

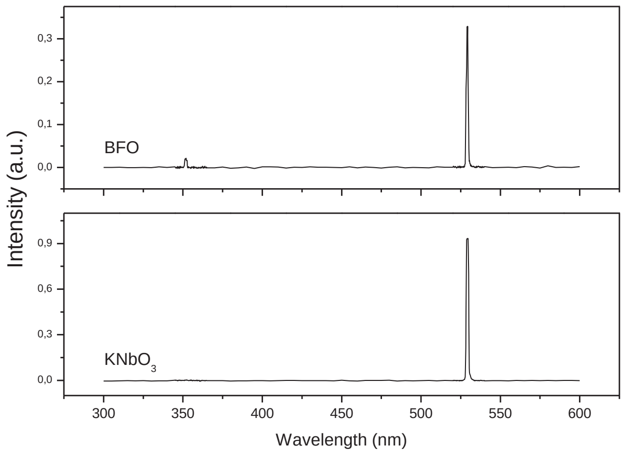

Figure S1. Emission spectra for BFO and KNbO3 nanoparticle ensembles by HRS.

2- Estimation of the average third-order susceptibility $<\chi^{(3)}>$

According to our previous work[1], the SH signal scattered from a nanocrystal suspension can be expressed as:

$$SH = G_{2\omega}.\frac{1}{\lambda_{2\omega}^4}.N.T_{2\omega}.\langle(\chi^{(2)})^2\rangle.V^2.I_\omega^2 \quad \textbf{(S1)}$$

$G_{2\omega}$ includes the fundamental constants that arise in the radiating field of an oscillating dipole and the experimental light collection efficiency at 2ω .N is the nanoparticle concentration, $T_{2\omega}$ an internal field factor, $\chi^{(2)}$ the second order susceptibility and V the nanoparticle volume. The internal field factor is introduced here to estimate the incident (macroscopic) optical field in the nanocrystal is given by:

$$T_{2\omega} = (t_\omega^2.t_{2\omega})^2 \text{ with } t_{\omega_i} = \frac{3n_s^2(\omega_i)}{2n_s^2(\omega_i)+3n_p^2(\omega_i)} \quad \textbf{(S2)}$$

$n_s^2(\omega_i)$ and $n_p^2(\omega_i)$ are the solvent and nanoparticle refractive indexes at ω_i, respectively.
In regard to the scattered TH signal, it can be expressed by using the same formalism:

$$TH = \frac{1}{4}.G_{3\omega}.\frac{1}{\lambda_{3\omega}^4}.N.T_{3\omega}.\langle(\chi^{(3)})^2\rangle.V^2.I_\omega^3 \quad \textbf{(S3)}$$

Here, $T_{3\omega}$ stands for $(t_\omega^3.t_{3\omega})^2$ and the $\frac{1}{4}$ factor originates from the definition of the nonlinear polarization as in the convention used by Bossard et al[2].
From the previous equations, the SH to TH ratio becomes:

$$\frac{SH}{TH} = 4.\frac{\lambda_{3\omega}^4}{\lambda_{2\omega}^4}\frac{G_{2\omega}}{G_{3\omega}}\frac{T_{2\omega}}{T_{3\omega}}\frac{\langle(\chi^{(2)})^2\rangle}{\langle(\chi^{(3)})^2\rangle}\frac{1}{I_\omega} \quad \textbf{(S4)}$$

$\frac{SH}{TH}.\frac{G_{3\omega}}{G_{2\omega}}$ which is given in the main text (*i.e.* 30 for BFO and 322 for $KNbO_3$) corresponds to the SH to TH ratio corrected for the different collection efficiencies at 2ω and 3ω,. I_ω was estimated at 11 GW/cm² and $\frac{T_{2\omega}}{T_{3\omega}}$ was calculated from the refractive index values taken in ref [3] for $KNbO_3$ and in ref [4] for BFO. Finally, we used for each nanocrystal our previous measured values of the orientation averaged second order susceptibility$\langle\chi^{(2)}\rangle$, namely $\langle d\rangle = 80\,pm/V$ for BFO[5] and $\langle d\rangle = 8.3\,pm/V$ for $KNbO_3$[1] with $\chi^{(2)} = 2d$. The same batches of $KNbO_3$ and BFO nanoparticles have been used in this study.
Following this approach, we estimated $\langle\chi^{(3)}\rangle \sim 1\ 10^{-19}\,m^2/V^2$ for $KNbO_3$ and $\langle\chi^{(3)}\rangle \sim 5.5\ 10^{-18}\,m^2/V^2$ for BFO.

References

1. Joulaud, C. *et al.* Characterization of the nonlinear optical properties of nanocrystals by Hyper Rayleigh Scattering. *J. Nanobiotechnology* **11,** S8 (2013).
2. Bosshard, C., Gubler, U., Kaatz, P., Mazerant, W. & Meier, U. Non-phase-matched optical third-harmonic generation in noncentrosymmetric media: cascaded second-order contributions for the calibration of third-order nonlinearities. *Phys. Rev. B* **61,** 10688 (2000).
3. Umemura, N., Yoshida, K. & Kato, K. Phase-matching properties of KNbO3 in the mid-infrared. *Appl Opt* **38,** 991–994 (1999).
4. Kumar, A. *et al.* Linear and nonlinear optical properties of BiFeO[sub 3]. *Appl. Phys. Lett.* **92,** 121915 (2008).
5. Schwung, S. *et al.* Nonlinear optical and magnetic properties of BiFeO3 harmonic nanoparticles. *J. Appl. Phys.* **116,** 114306 (2014).
